# Modeling retinitis pigmentosa through patient-derived retinal organoids

**DOI:** 10.1016/j.xpro.2021.100438

**Published:** 2021-04-08

**Authors:** Yan-Ping Li, Wen-Li Deng, Zi-Bing Jin

**Affiliations:** 1 Beijing Institute of Ophthalmology, Beijing Tongren Eye Center, Beijing Tongren Hospital, Capital Medical University, Beijing Ophthalmology & Visual Sciences Key Laboratory, Beijing, 100730 China; 2 School of Ophthalmology and Optometry, The Eye Hospital, Wenzhou Medical University, Wenzhou 325027 China; 3 The First Affiliated Hospital of Chongqing Medical University, Chongqing Key Laboratory of Ophthalmology, and Chongqing Eye Institute, Chongqing 400016, China

**Keywords:** Cell isolation, CRISPR, Stem Cells, Cell Differentiation, Organoids

## Abstract

Human-induced pluripotent stem cells (hiPSCs) can be differentiated into well-structured retinal organoids. In this protocol, we successfully established 3D retinae from patient-derived hiPSCs and built the retinitis pigmentosa model *in vitro*. Moreover, mutation in the retinitis pigmentosa GTPase regulator (RPGR) gene was corrected by CRISPR-Cas9 gene editing, which rescued the structure and function of the 3D retinae.

For complete details on the use and execution of this protocol, please refer to Deng et al. (2018).

## Highlights

• Patient-specific human induced pluripotent stem cells (hiPSCs) were established.
• The *RPGR* mutation was corrected by CRISPR/Cas9 in hiPSCs.
• hiPSCs were differentiated into 3D retinal organoids.

## Graphical abstract

**Figure:**
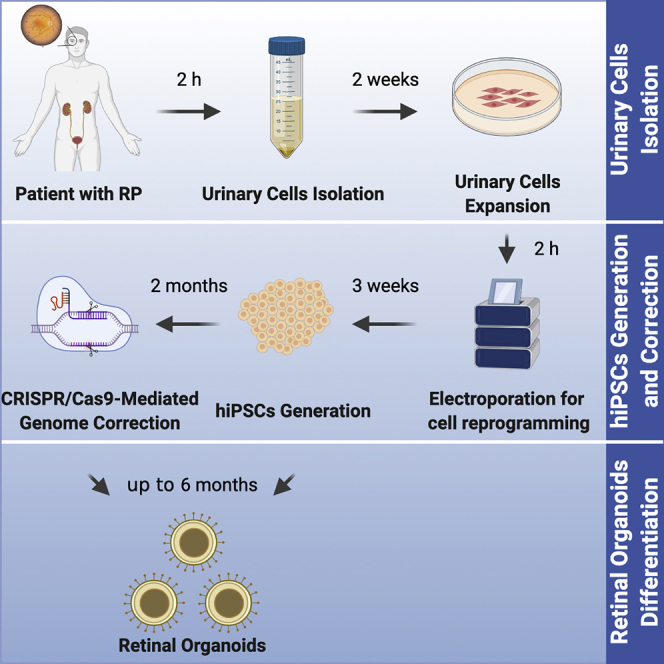

## Teaser

Human-induced pluripotent stem cells (hiPSCs) can be differentiated into well-structured retinal organoids. In this protocol, we successfully established 3D retinae from patient-derived hiPSCs and built the retinitis pigmentosa model *in vitro*. Moreover, CRISPR-Cas9-mediated correction of the retinitis pigmentosa GTPase regulator mutation rescued the structure and function of the 3D retinae.

## Before you begin

### Reconstitution of reagents

**Timing: 2.5–3.0 h**

1. 5 mg/mL DNase I (100×)
  a. Dissolved 15 mg of DNase I in 3 mL of ultra-pure water.
  b. After mixing well, use a 0.22-μm membrane filter for the sterilizing filtration.
  c. Aliquot 100 μL of the dissolved DNase I into sterile 200-μL Eppendorf tubes.
  d. Store the reconstituted solution at −20°C, which will be stable until the expiration date printed on the label.
2. 10 mM Y-27632 (1,000×)
  a. Centrifuge quickly before opening the lid, 2000 × *g* for 30 s, to ensure that all the powder sediments at the bottom of the tube.
  b. Dissolved 10 mg of Y-27632 in 3.1226 mL of dimethyl sulfoxide (DMSO).
  c. After complete dissolution, aliquot 20 μL of dissolved Y-27632 into sterile 200-μL Eppendorf tubes.
  d. Store the reconstituted solution at −80°C where it can remain for up to two years, and the powder can be stored at −20°C for three years.
3. Recombinant human BMP4 (hBMP4)
  a. Add 10 mg of bovine serum albumin (BSA) powder to 10 mL of ultra-pure water.
  b. Mix slowly for 30 min, at least, at 25°C until the BSA powder is completely dissolved.
  c. Filter the solution using a 0.22-μm filter to ensure sterile filtration.
  d. Centrifuge quickly at 2000 × *g* for 30 s, before opening the lid, to ensure that all the powder sediments at the bottom of the tube.
  e. Dissolved 50 μg of hBMP4 in sterile 4-mM HCl containing 0.1% BSA.
  f. After complete dissolution, aliquot 20 μL of dissolved hBMP4 into sterile 200-μL Eppendorf tubes.
  g. Store the solution at −20°C for up to 6 months. After reconstitution, it can be stored at 2°C to 8°C for up to 1 month, or store at −70°C for up to 3 months.
4. 5 mM retinoic acid (20×)
  a. Dissolve 100 mg of retinoic acid in 3.33 mL of DMSO to obtain a stock solution (100 mM).
  b. Aliquot and store in light protected vials at −80°C for up to 2 weeks.
  c. Dilute 100 mM retinoic acid using DMSO to a 5-mM concentration and store the solution in the dark at -20°C for up to 2 weeks.
  d. Diluted it in tissue culture medium right before use.

**CRITICAL:** Retinoic acid is more sensitive to light, heat, and air in solution. The prepared retinoic acid stock solution should be stored in the dark until use.

5. 100 mM Taurine (1,000×)
  a. Add 0.125 g of taurine in to 10 mL of Dulbecco's phosphate buffered saline (DPBS).
  b. After complete dissolution, aliquot 1 mL of taurine solution into sterile 1.5-mL Eppendorf tubes.
  c. Store the solution at −20°C until the expiration date printed on the label.
6. Matrigel preparation (protein concentration: 8.1 mg/mL)
  a. Thaw Matrigel at 4°C for 1–2 days until it melts completely.
  b. Store the 1.5-mL Eppendorf tubes and pipettes in the −20°C freezer one day in advance to prechill them.
  c. Aliquot 370 μL of the Matrigel into prechilled 1.5-mL Eppendorf tubes on ice using prechilled pipettes.
  d. Store the aliquots quickly at −20°C after labeling them.

**CRITICAL:** Freeze-thaw cycles should be minimized by aliquoting into one-time-use aliquots. Store aliquots at −20°C in the freezer until use.

### Preparation of tissue culture ware

**Timing: 1–2 h**

7. Matrigel coating (protein concentration: 8.1 mg/mL)
  a. Thaw 370 μL of aliquots at 4°C for 1–2 days until it melts completely.
  b. Add 1 aliquot of Matrigel to 24 mL of Dulbecco's Modified Eagle's medium DMEM/F12 at 4°C using prechilled pipettes and mix well.
  c. Add 1 mL of a Matrigel solution to each well of 6-well tissue culture plates and incubate for 30–60 min at 37°C before use.
8. Gelatin coating
  a. Add 1 mL of 0.1% gelatin to each well of the 6-well tissue culture plates.
  b. Cover the entire bottom surface of plate after gentle shaking.
  c. Incubate it for 30 min at 37°C before use.

## Key resources table

**Table:** 

REAGENT or RESOURCE	SOURCE	IDENTIFIER
**Antibodies**		

PAX6 antibody	BioLegend	Cat# 901301
CRX antibody	Abnova	Cat# H00001406-M02
Rhodopsin	Sigma	Cat# O4886
Recoverin	Millipore	Cat# AB5585
L/M-opsin	Millipore	Cat# AB5405
ARL13B	ProteinTech	Cat# 17711-1-ap
DAPI	Invitrogen Antibodies	Cat# D-1306
Donkey anti-rabbit 594	Invitrogen Antibodies	Cat# A-21207
Donkey anti-rabbit 488	Invitrogen Antibodies	Cat# A-21206
Donkey anti-mouse 594	Invitrogen Antibodies	Cat# A-21203
Donkey anti-mouse 488	Invitrogen Antibodies	Cat# A-21202

**Chemicals, peptides, and recombinant proteins**		

GlutaMAX	Life Technologies	Cat# 35050-061
MEM non-essential amino acid solution (100×) (NEAA)	Sigma	Cat# M7145
Fetal bovine serum (FBS)	Biological Industries	Cat# 04-002-1A
AlbuMAX II Lipid-Rich BSA	Gibco	Cat# 11021037
Primocin	InvivoGen	Cat# ant-pm-1
Penicillin-streptomycin (PS)	Gibco	Cat# 15140-122
Dimethyl sulfoxide (DMSO)	Sigma	Cat# D2650
TrypLE Select (1×), no phenol red	Life Technologies	Cat# 12563-011
Accutase	STEMCELL Technologies Inc	Cat# 07920
DNase I	Roche	Cat# 11284932001
Y-27632-2HCl	Selleck	Cat# S1049
KnockOut Serum Replacement - Multi-Species (KSR)	Gibco	Cat# A3181502
Chemically Defined Lipid Concentrate	Thermo	Cat# 11905031
Monothioglycerol	Sigma	Cat# M6145
Recombinant human BMP4	R&D Systems	Cat# 314-BP
N-2 Supplement (100×), liquid	Life Technologies	Cat# 17502-048
Retinoic acid (RA)	Sigma	Cat# R2625
Taurine	Sigma	Cat# T8691
Matrigel, Growth Factor Reduced (GFR) Basement Membrane Matrix, Phenol Red-Free, ∗LDEV-Free	Corning	Cat# 356231
EmbryoMax 0.1% Gelatin Solution	Millipore	Cat# ES-006-B
G418 disulfate salt	Sigma	Cat# G1279
Agarose, low gelling temperature	Sigma-Aldrich	Cat# A0701

**Critical commercial assays**		

DMEM/Ham’s F12	Gibco	Cat# 10565-042
Ham’s F12	Gibco	Cat# 11765-054
DMEM basic	Gibco	Cat# C11995500bt
Dulbecco's phosphate buffered saline (DPBS)	Gibco	Cat# C141905005BT
TeSR-E8 Kit for hESC/hiPSC Maintenance	STEMCELL Technologies	Cat# 05990
ncEpic hPSC Medium	Nuwacell Biotechnologies Co., Ltd	Cat# RP01001
Iscove’s Modified Dulbecco Medium (IMDM)	Gibco	Cat# 12440053
Ham's F-12 Nutrient Mixture	Gibco	Cat# 11765-054
REGM BulletKit	Lonza	Cat# CC-3190
P3 Primary Cell 4D-Nucleofector X Kit L	Lonza	Cat# V4XP-3024
Cultured Cells DNA Kit	Simgen	Cat# 3001250
Phanta Super-Fidelity DNA Polymerase	Vazyme	Cat# P505-d3
2× power Taq PCR Master Mix	BioTeke	Cat# PR1702
P3 Primary Cell 4D-Nucleofector X Kit	Lonza	Cat# V4XP-3024
QIAquick Gel Extraction Kit (250)	QIAGEN	Cat# 28706
Endo-free Plasmid Mini Kit I (200)	Omega	Cat# D6948-02
pEASY-Blunt Simple Cloning Kit	TransGen Biotech	Cat# CB111-01

**Oligonucleotides**		

Primer: pX330-sgRNA-F 5′- CACCGC ATGTAAACAACGTGTCACAA -3′	This paper	N/A
Primer: pX330-sgRNA-R 5′- AAACTTG TGACACGTTGTTTACATGC -3′	This paper	N/A
Primer F for correction verification CACAGACTAGAGAGTGGCAC	This paper	N/A
Primer R for correction verification CCTCTACCCTTGTCTTTCTC	This paper	N/A

**Recombinant DNA**		

pX330 plasmid	Addgene	Cat# 42230
Episomal reprogramming plasmids	System Biosciences (SBI)	Cat# SC900A-1

**Software and algorithms**		

Leica software	Leica	http://www.leica-microsystems.com/home/
CRISPR sgRNA design tool	CRISPOR	http://crispor.tefor.net/

**Other**		

6-well plates	Cyagen	Cat# 40106
Non-stick 10-cm petri dish	Greiner	Cat# 663102
96-well V-bottomed conical wells	Sumitomo Bakelite	Cat# MS-9096VZ
1,000-μL Pipette tips	Axygen	Cat# T-1000-R-S
200-μL Universal Fit Pipet Tip	Axygen	Cat# T-200-Y-R-S
10-μL Microvolume Pipet Tips	Axygen	Cat# T-300-R-S
1.5 mL EP	Axygen	Cat# MCT-150-C
0.6 mL EP	Axygen	Cat# MCT-060-C
0.2 mL EP	Axygen	Cat# PCR-02-C
15-mL Centrifuge tube	BD Falcon	Cat# 352097
50-mL Centrifuge tube	BD Falcon	Cat# 352070
5-mL Pipetting tube	BD Falcon	Cat# 357543
10-mL Pipetting tube	BD Falcon	Cat# 357551
25-mL Pipetting tube	BD Falcon	Cat# 357525
1,000-μL Pipette tips	Axygen	Cat# T-1000-R-S
200-μL Pipette tips	Axygen	Cat# T-200-Y-R-S
10-μL pipette tips	Axygen	Cat# T-300-R-S
Cryopreservation tubes	Corning	Cat# 430488
Sterile square media bottle	Nalgene	Cat# c0006558
Millex-GP, 0.22-μm filter	Millpore	Cat# SLGP033RB
1-mL Injection needle	Kangkang	N/A
50-mL Injection needle	Kangkang	N/A
V-Lance knife	Alcon Surgical	Cat# 8065912001
Counting chambers	RONGYI	Cat# 1103
4°C freezers	Haier	Cat# HXC-936
−20°C freezers	Haier	Cat# BCD-256WDGK
−80°C freezers	Panasonic	Cat# MDF-U3386S
CO_2_ incubator	Thermo Scientific	Cat# 3111
Water bath	Yiheng	Cat# DK-8AB
Thermal cycler	Life Technologies	Cat# 4483636
Centrifuge	Eppendorf	Cat# 5702
Liquid nitrogen storage dewar	Thermo Scientific	Cat# CY50985
Class II, Type A2 Biosafety Cabinets	Thermo Scientific	Cat# 1300 Series A2
4D-Nucleofector Core Unit	Lonza	Cat# AAF-1002B
4D-Nucleofector X Unit	Lonza	Cat# AAF-1002X
Microscope	Life Technologies	Cat# EVOS XL

## Materials and equipment

### Reconstitution of the media

***Note:*** Make sure all the media and reagents are prepared under aseptic conditions and warmed at room temperature (20°C–25°C) before use.

### Medium reconstitution for urinary cells

**Table:** Primary medium

Reagent	Final concentration	Amount
DMEM	44.5%	222.5 mL
Ham’F12	44.5%	222.5 mL
FBS	10%	50 mL
Penicillin-Streptomycin (PS)	1% (100 U/mL)	5 mL
**Total**	**n/a**	**500 mL**

***Note:*** Store the primary medium in the dark at 4°C and use it within 2 weeks.

**Table:** Proliferation medium: (Renal Epithelial Basal Medium (REBM) Bullet Kit reconstitution)

Reagent	Final concentration	Amount
Renal Epithelial Basal Medium (REBM)	n/a	500 mL
SingleQuots Kit	1.2%	6 mL
**Total**	**n/a**	**506 mL**

***Note:*** Store the proliferation medium in the dark at 4 °C and use it within 2 weeks.

**Table:** Washing buffer:

Reagent	Final concentration	Amount
Dulbecco's Phosphate Buffered Saline (DPBS)	n/a	494 mL
Primocin	0.2%	1 mL
PS	1% (100 U/mL)	5 mL
**Total**	**n/a**	**500 mL**

***Note:*** The solution should be freshly reconstituted before use.

**Table:** 100 μL Nucleofection solution

Reagent	Final concentration	Amount
4D-Nucleofector solution	82%	82 μL
Supplement solution	18%	18 μL
**Total**	**n/a**	**100 μL**

***Note:*** The solution should be freshly reconstituted before use.

### Medium reconstitution for hiPSCs

**Table:** 0.5-mM EDTA solution

Reagent	Final concentration	Amount
0.5 M EDTA	1% (0.5 mM)	500 μL
DPBS	n/a	49.5 mL
**Total**	**n/a**	**50 mL**

***Note:*** Store the solution at 4°C for up to 1 month.

**Table:** TeSR-E8 medium

Reagent	Final concentration	Amount
TeSR-E8 Basal Medium	n/a	480 mL
TeSR-E8 25X Supplement	4% (1×)	20 mL
**Total**	**n/a**	**500 mL**

***Note:*** Store the medium at 4°C for up to 1 month.

**Table:** ncEpic hPSC medium

Reagent	Final concentration	Amount
ncEpic Basal Medium	n/a	496 mL
ncEpic 125X Supplement	0.8% (1×)	4 mL
**Total**	**n/a**	**500 mL**

***Note:*** Store the medium at 4°C and use it within 2 weeks.

**Table:** hiPSCs cryopreservation solution:

Reagent	Final concentration	Amount
TeSR-E8	90%	900 μL
DMSO	10%	100 μL
**Total**	**n/a**	**1 mL**

***Note:*** The solution should be freshly reconstituted before use.

### Medium reconstitution for retinal organoid differentiation

**Table:** Cell dissociation solution:

Reagent	Final concentration	Amount
TrypLE Select	n/a	1 mL
DNase I (5 mg/mL)	0.05 mg/mL	10 μL
Y-27632 (10 mM)	20 μM	2 μL
**Total**	**n/a**	**1.012 mL**

**Note:** The solution should be freshly reconstituted before use.

**Table:** Differentiation medium:

Reagent	Final concentration	Amount
IMDM	44%	22 mL
F12	44%	22 mL
KSR	10%	5 mL
Glutamax	1%	500 μL
Monothioglycerol	450 μM	1.95 μL
PS	1% (100 U/mL)	500 μL
**Total**	**n/a**	**50 mL**

***Note:*** Store the differentiation medium at 4°C for up to 1 month.

**Table:** Neural retina medium:

Reagent	Final concentration	Amount
DMEM/F12	n/a	439.45 mL
FBS	10%	50 mL
N-2 Supplement (100 X)	1% (1 X)	5 mL
Retinoic acid (RA)	0.5 μM	50 μL
Taurine	0.1 mM	500 μL
PS	1% (100 U/mL)	5 mL
**Total**	**n/a**	**500 mL**

***Note:*** Store the medium in the dark at 4°C and use it within 2 weeks.

## Step-by-step method details

### Establishment of hiPSCs from urine samples

***Note:*** All the previously described procedures (Zhou et al., 2012) for urinary cell isolation and expansion were used with some modifications (Deng et al., 2018).

#### Urinary cell isolation

**Timing: 2 h**

1. Collect mid-streams urine samples (100 mL–300 mL) from volunteers.

**CRITICAL:** Discard the first streams of urine.

**CRITICAL:** The urine container must be sterile, disposable sterile bottles are recommended.

**CRITICAL:** It is also recommended that the opened container should not contact volunteers’ skin to prevent contamination.

2. Transfer them into 50-mL tubes using a 25-mL pipette as soon as possible.

**CRITICAL:** Urinary cells should be isolated immediately after urine collection.

3. Centrifuge the samples at 400 × *g* for 10 min at room temperature (20°C–25°C).
4. Discard the supernatant carefully until 1 mL or less of the liquid is left in the tube.
5. Resuspend and collect the liquid from one sample into a 50-mL tube.
6. Add 10 mL of washing buffer to dilute the samples.
7. Centrifuge at 200 × *g* for 10 min at room temperature (20°C–25°C).
8. During centrifugation, aspirate the gelatin solution of the cell culture plates and wash twice using washing buffer.
9. Add 1 mL of the primary medium into each gelatin-coated well.
10. After centrifugation, discard the supernatant until 0.2 mL or less of the sample is left in the tube.
11. Resuspend the sediment in 1 mL of primary medium.
12. Transfer the cells onto gelatin-coated 6-well plates and then incubate the plates.

**CRITICAL:** Steps 9–12 should be performed in the dark.

**CRITICAL:** The gelatin-coated plates should be freshly coated; thus, it would better to coat the plates 0.5–1 h before use.

#### Expansion of urinary cells

Day 1, day 2, and day 3: Addition of the primary medium

**Timing: 0.5 h**

13. Add 1 mL of the primary medium to the wells to maintain the concentration of antibiotics and nutrition.

**CRITICAL:** Step 13 should be performed in the dark.

Day 4: Medium change to the proliferation medium

**Timing: 0.5 h**

**Figure 1:**
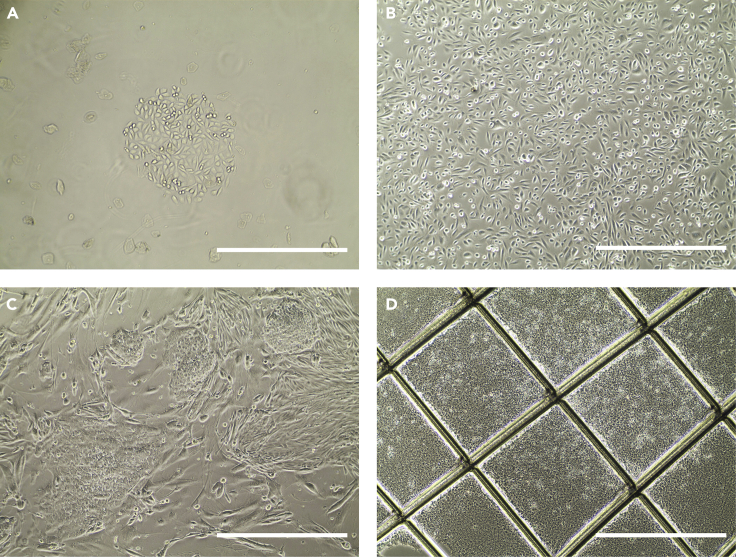
Establishment of hiPSCs from urine samples Bright field images are shown in (A–D). (A) Urinary cells at day 4 after collection. (B) Urinary cells at day 3 after passaging. (C) hiPSC colonies at day 5 after reprogramming. (D) Breaking the hiPSC colonies into a checkerboard-shaped grid before isolation. Scale bars, 400 μm.

14. Aspirate the culture medium until 1 mL is left in the wells.
15. Add 1 mL of the proliferation medium. The urinary cell colonies can be seen under an inverted microscope (Figure 1A).

**CRITICAL:** Steps 14 and 15 should be performed in the dark.

The following days: Half medium change using the proliferation medium

**Timing: 0.5 h**

16. Change half of the proliferation medium daily.
17. Aspirate 1 mL of the medium in the wells, and add 1 mL of the proliferation medium.

**CRITICAL:** Steps 16 and 17 should be performed in the dark.

#### Passaging the urinary cells

**Timing: 0.5 h**

18. Passage the urinary cells when they reach 80%–90% confluence.
19. Aspirate all the culture medium, and wash the cells twice using 1 mL of DPBS each time.
20. Then, dissociate the cells by adding 0.5 mL of TrypLE Select to each well and incubate the plates for 5 min at 37°C.
21. Add the medium containing 10% FBS to stop the reaction.
22. Centrifugate cells at 200 × *g* for 5 min and resuspend them using the proliferation medium after the supernatant is completely removed.
23. Finally, seed the urinary cells in new gelatin-coated plates at a 1:4 split ratio for further expansion (Figure 1B).

***Note:*** Small urinary cell colonies appeared within 3–5 days after plating and grew steadily.

**CRITICAL:** The gelatin-coated plates should be freshly coated; thus, it would better to coat the plates 0.5–1 h before use.

#### Generation of integration-free hiPSCs using episomal plasmids

**Timing: 1 h**

24. Add the reprogramming plasmids (2 μg) encoding Oct4, Sox2, Lin28, Klf4, L-myc, p53shRNA, and the miR-302/367 cluster to the nucleofection solution.
25. Wash the urinary cells twice with DPBS and then dissociate them to single cells using 0.5 mL of TrypLE Select.

**CRITICAL:** The cells used for transfection were in a single cell suspension: nucleofection clumps led to a low transfection efficiency.

26. Count the cells using a counting chamber, and aspirate 10^5^–10^6^ cells for nucleofection.

**CRITICAL:** A total of 10^5^–10^6^ cells from each sample are required for transfection, since a lower or higher number of cells might decrease the transfection efficiency.

27. Then, centrifuge cells at 200 × *g* for 3.5 min at room temperature (20°C–25°C).
28. Aspirate the supernatant as much as possible by using pipette tips.
29. Resuspend cells in 100 μL of nucleofection solution.
30. Gently transfer the sample to Nucleocuvette vessels and tap the vessels to make sure the sample is at the bottom of the vessels.

**CRITICAL:** Tap the Nucleocuvette vessels gently after the mixture is added, to avoid the presence of air bubbles and to completely cover the bottom of the cuvette with the sample.

31. Transfer the vessels to the retainer and electroporate them using the 4D-nucleofector system by using the T-020 program. Troubleshooting 1
32. Finally, after the electroporation procedure, gently transferred the urinary cells into Matrigel-coated plates containing TeSR-E8 medium.

**CRITICAL:** The Matrigel-coated plates should be freshly coated; thus, it would better to coat the plates 0.5–1 h before use.

33. Change the medium after 24 h of transfection.
34. Grow the transfected urinary cells in a hypoxia chamber (5%–6% O_2_) until the human ESC-like colonies appear (Figure 1C).

#### hiPSC colony isolation and expansion

**Timing: 1 h**

35. Approximately three weeks post-electroporation, isolate hiPSC colonies according to following steps. First, break the hiPSC colonies into a checkerboard-shaped grid by using sharp needles under an inverted microscope (Figure 1D). Troubleshooting 2

**Figure 2:**
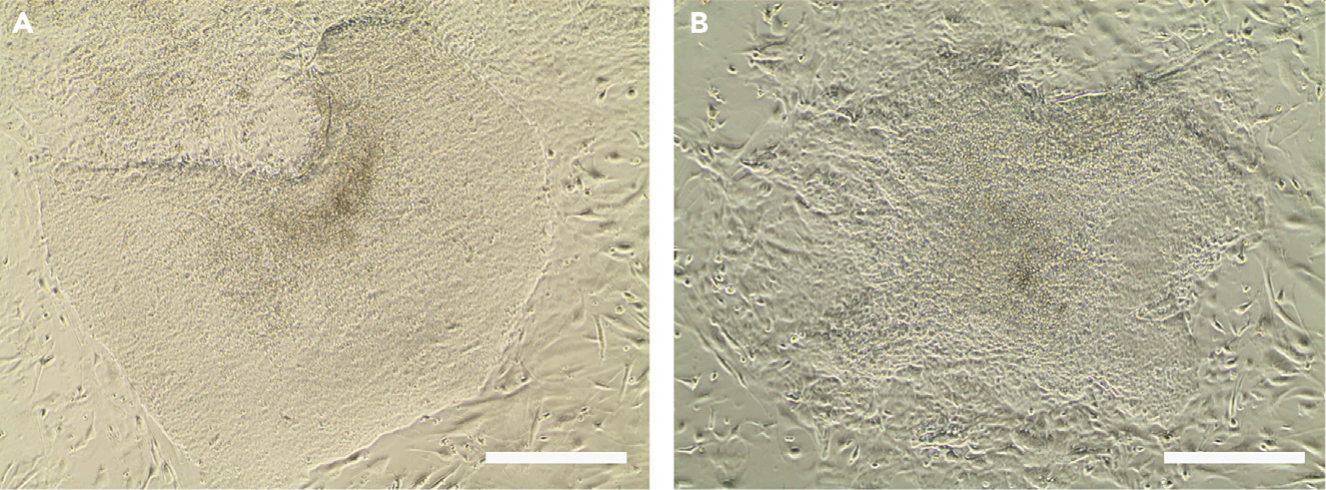
Representative pictures of "good" and "bad" colonies Bright field images are shown in (A and B). (A) “Good" colony after reprogramming. (B) “Bad" colony after reprogramming. Scale bars, 400 μm.

**CRITICAL:** Pick only well-separated and hESC-like colonies to make sure they are clones. Representative pictures of "good" colonies and "bad" colonies are shown in Figure 2.

36. Then, collect the colony pieces using 100-μL pipette tips and transfer them to new Matrigel-coated plates containing fresh TeSR-E8 medium supplemented with 10 μM Y-27632.

**CRITICAL:** One colony should be seeded per well. Each time, when a colony is picked, the needle and tip should be replaced.

**CRITICAL:** The Matrigel-coated plates should be freshly coated; thus, it would better to coat the plates 0.5–1 h before use.

### CRISPR-Cas9-mediated genome editing

#### sgRNA design and cloning

**Timing: 2 weeks**

37. *RPGR* mutation is used for hiPSC correction (Figure 3). Use the CRISPR sgRNA design tool to design sgRNA target exon 14 near the mutation site.

**Figure 3:**
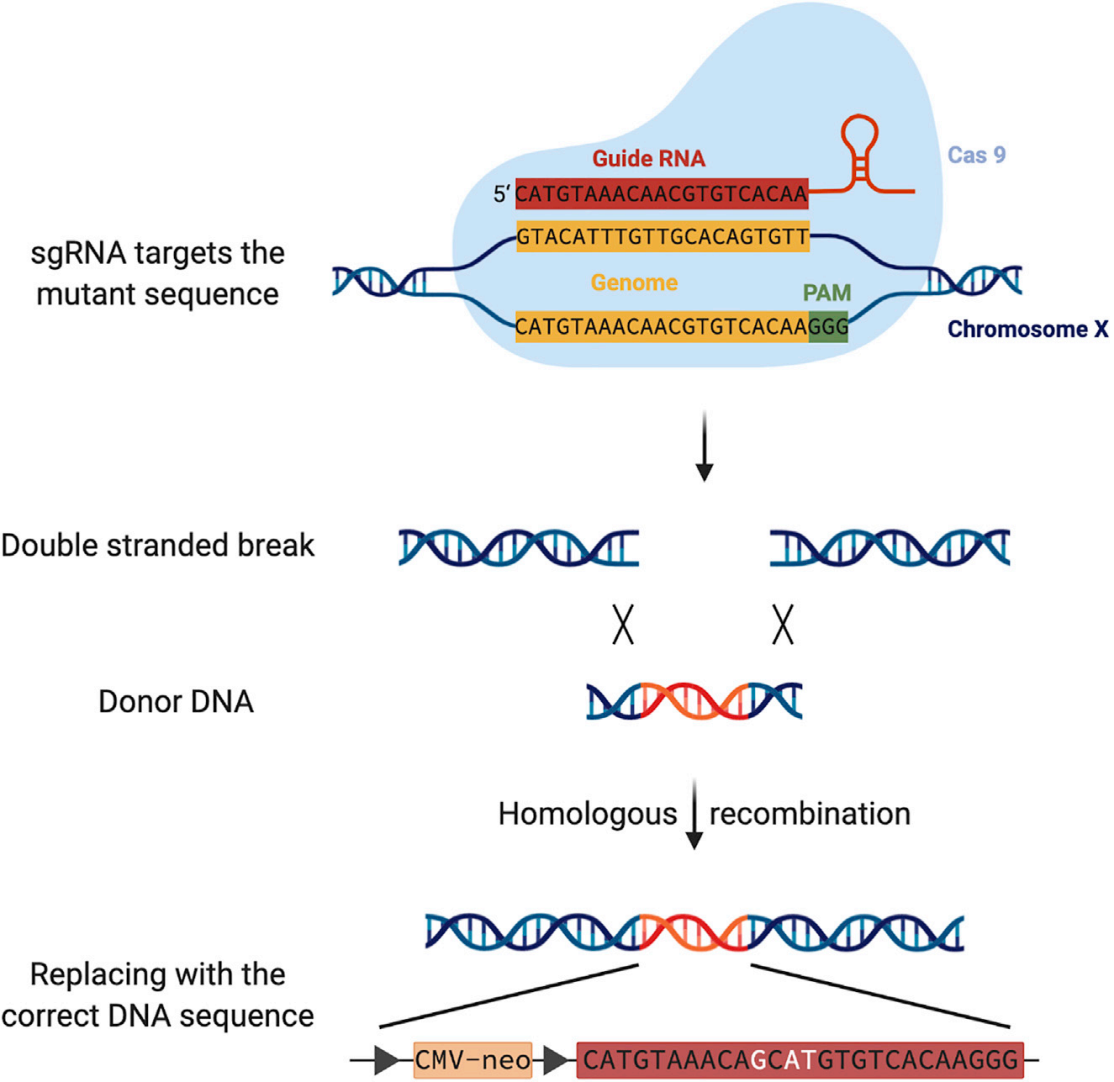
Gene editing using CRISPR-Cas9 by using homology-directed repair
38. Dissolve both oligos to 100 μM using nuclease-free water.
39. Phosphorylate and anneal the two oligos using the following conditions: 37°C for 30 min, 95°C for 5 min and then bring the temperature down to 25°C at 5°C/min.

**CRITICAL:** Set the thermal cycler’s heated lid to 75°C to prevent samples from evaporating and drying out.

40. In the meantime, digest the pX330 plasmid using BbSI at 37°C for 30 min.

***Alternatives:*** Water bath can be used instead of the thermal cycler for heating.

41. Purify the digested pX330 plasmid using a 1% agarose gel and QIAquick Gel Extraction Kit, following the manufacturer’s instructions.

**CRITICAL:** 1% agarose gel should be freshly reconstituted before use.

42. Detect the concentration of the digested pX330 plasmid using NanoDrop device.
43. Then, set up a ligation reaction and incubate the digested pX330 plasmid and sgRNA at room temperature (20°C–25°C) for 10 min.
44. Transform the product of ligation into competent cells following the manufacturer’s instructions.

**CRITICAL:** The product of the ligation reaction should be stored at −20°C or on ice before use.

**CRITICAL:** After transformation, pick only well separated and round colonies to make sure they are clones.

**CRITICAL:** Keep the Luria-Bertani (LB) agar plate containing colonies at 4°C for no more than one month after sealing, in order be able to isolate more colonies, if necessary.

45. After amplification, extract the plasmid DNA containing the target sequence using QIAGEN EndoFree Plasmid Maxi Kit, by following the manufacturer’s instructions.
46. Store it at −20°C before use.
47. Analyze the plasmid DNA using Sanger sequencing.

**Pause point:** The plasmid DNA may be safely stored in the freezer until convenient.

**CRITICAL:** The plasmid DNA can be store at −20°C for up to 6 months, but freeze-thaw cycles should be avoided.

#### Donor DNA design and plasmid cloning

**Timing: 2 weeks**

48. As a homologous recombinant template, amplify donor exon 14 from the normal template. Homology arms and a selection cassette are recommended.
49. After amplified and purified, insert a 3.4 kbp homology directed repair (HDR) fragment carrying a selection cassette of neomycin (G418) into pEASY-Blunt simple cloning vector following pEASY-Blunt cloning protocol.

**CRITICAL:** After transformation, pick only well separated and round colonies to make sure they are clones.

**Pause point:** Maintain the LB agar plate containing colonies at 4°C for no more than one month after sealing, in order to be able to isolate more colonies, if necessary.

50. Analyze the colonies using Sanger sequencing.
51. After amplification, extract the plasmid DNA by using EndoFree Plasmid Mini Kit, following the manufacturer’s instructions.
52. Store the plasmid DNA at −20°C before use.

**Pause point:** The plasmid DNA may be safely stored in the freezer until convenient.

#### Plasmid electroporation

**Timing: 1 hour**

53. When the hiPSCs show 70%–80% confluence, use accutase to dissociate hiPSCs into single cells.
54. Harvest and count the cells, centrifuge the required number of cells, and completely remove the supernatant.
55. For the genome correction knock-in, mix 2 μg of the constructed pX330 plasmid and 2 μg of the targeting vector in nucleofector solution before electroporation.
56. Resuspend the required number of cells in the nucleofector solution.

**CRITICAL:** A total of 2–3 × 10^5^ cells from each sample are required, since a lower or higher number of cells may decrease the transfection efficiency.

57. Carefully transfer the mixture to 100-μL Nucleocuvette vessels.

**CRITICAL:** Tap the Nucleocuvette vessels gently after the mixture is added, to avoid the presence of air bubbles and to completely cover the bottom of the cuvette with the sample.

58. Perform transfection using the 4D-nucleofector system, under program CA-137.
59. After electroporation, gently transfer the cells to Matrigel-coated plates containing TeSR-E8 medium supplemented with 10 μM Y-27632.

***Note:*** It has been reported that using Y-27632 may increase the viability of stem cells (Watanabe et al., 2007).

**CRITICAL:** The vessels should be carefully removed from the retainer after electroporation.

**CRITICAL:** The Matrigel-coated plates should be freshly coated; thus, it would better to coat the plates 0.5–1 h before use.

#### Neomycin selection

**Timing: 2 weeks**

60. When the transfected cells reach 70%–80% confluence, add G418 to TeSR-E8 medium at a final concentration of 200 μg/mL for cell selection.
61. Wash the cells with DPBS and refresh the medium daily.

#### Genome correction clone screening using PCR and sequencing

**Timing: 2 weeks**

#### Collect the DNA

62. After selection, G418-insensitive hiPSC colonies were isolated and expanded.
63. Remove the TeSR-E8 medium, rinse the hiPSCs twice with DPBS, and dissociate the cells using an EDTA solution for 5 min.
64. Remove the EDTA and resuspend hiPSCs in fresh TeSR-E8 medium.
65. Centrifuge the suspension at 200 × *g* for 5 min and remove the medium.

**Pause point:** Cells can be stored at −20°C for up to 6 months for subsequently genomic DNA isolation.

66. Then, extract the genomic DNA by using Cultured Cells DNA Kit, according to the manufacturer’s instructions.

**CRITICAL:** The DNA can be stored at −20°C for up to 6 months, but freeze-thaw cycles should be avoided.

#### PCR and production verification using sanger sequencing

67. Amplify the genomic DNA using a Super-Fidelity PCR Kit and the following parameters: denaturation for 30 s at 98°C, denaturation for 15 s at 98°C, annealing for 60 s at 60°C, and extension for 30 s at 72°C for 35 cycles.

**CRITICAL:** Set the thermal cycler’s heated lid to 75°C to prevent samples from evaporating and drying out.

68. Finally, perform an extension for 10 min at 72°C and hold the product at 4°C.
69. Verify the PCR product using Sanger Sequencing.

**Pause point:** The PCR product may be safely stored in the freezer until convenient.

**CRITICAL:** The final product of PCR can be stored at −20°C for up to 6 months, but freeze-thaw cycles should be avoided.

### Maintenance of hiPSC colonies

#### hiPSC recovery

**Timing: 0.5 h**

70. Remove the cell lines from liquid nitrogen and thaw them in a 37°C water bath as soon as possible. Troubleshooting 3

**CRITICAL:** Put the cell lines in water bath immediately after taking it out of the liquid nitrogen.

71. Then, slowly dilute them with TeSR-E8 medium in a new tube.
72. Centrifuge at 200 × *g* for 5 min at room temperature (20°C–25°C).
73. Aspirate the supernatant and resuspend cells in TeSR-E8 medium using gentle pipetting.
74. Then, culture cells in Matrigel-coated 6-well plates containing TeSR-E8 medium supplemented with 10 μM Y-27632, and incubate them after shaking.

**CRITICAL:** The Matrigel-coated plates should be freshly coated; thus, it would better to coat the plates 0.5–1 h before use.

***Alternatives:*** For hiPSCs maintenance, ncEpic hPSC Medium can be used instead of TeSR-E8 medium.

#### Medium change

**Timing: 0.5 h**

75. Aspirate the medium in the 6-well plates and wash the hiPSCs with DPBS once or twice.
76. Add 2–3 mL of TeSR-E8 medium to the plate and incubate them.

#### Passaging and cryopreservation of hiPSCs

**Timing: 0.5 h**

77. When cell confluence reaches 70% or more, passage the cells. Aspirate the medium in the 6-well plates and wash the hiPSCs with DPBS once.
78. Add 1 mL of EDTA to digest the hiPSCs at 37°C for 5 min.
79. For passaging, aspirate the EDTA and resuspend hiPSCs in TeSR-E8 Medium containing 10 μM Y-27632.

***Note:*** It has been reported that using Y-27632 may increase the viability of stem cells (Watanabe et al., 2007).

80. Break hiPSC clumps into smaller pieces using gentle pipetting, and add an appropriate volume of cells into a new Matrigel-coated 6-well plate.

**CRITICAL:** The hiPSC used for passaging should not in a single cell suspension, single cell led to a poor pluripotency status.

81. Passage the cells every third or fourth day.
82. For cell freezing, aspirate the EDTA (from step 78) and resuspend hiPSCs with cell cryopreservation solution.
83. Transfer hiPSCs in a freezing tube and perform gradient cooling.
84. Store the hiPSCs in liquid nitrogen until use.

**Pause point:** The hiPSCs can be stored in liquid nitrogen until use.

### Generation of retinal organoids from hiPSCs

***Note:*** All the previously described procedures (Kuwahara et al., 2015) for retinal organoid differentiation were used with slight modifications (Gao et al., 2020; Liu et al., 2020).

#### Day 0: Reaggregate cells

**Timing: 1 h**

85. When cell confluence surpasses 70%, dissociate the hiPSC colonies using a cell dissociation solution at 37°C for 3.5 min. Troubleshooting 4
86. Then resuspend them in the differentiation medium.
87. Break the hiPSC clumps into single cells using gentle pipetting.
88. Count and dilute cells in the differentiation medium supplemented with 20 μM Y-27632.
89. Then, reaggregate cells in 96 V-bottomed conical wells at a density of 12,000 cells per well, in a volume of 100 μL.
90. Incubate the plates after shaking.

#### Day 6: Addition of hBMP4

**Timing: 0.5 h**

91. To change the differentiation medium, aspirate 100 μL of the medium from each well.
92. Add 100 μL of the differentiation medium supplemented with 20 μM Y-27632 and 1.5 nM (55 ng/mL) hBMP4.
93. Incubate the plates in CO_2_ incubator.

#### Day 9: Half differentiation medium changes

**Timing: 0.5 h**

94. Change half of the differentiation medium to achieve a lower concentration of hBMP4 (0.75 nM; final concentration: 27.5 ng/mL).
95. Aspirated 60 μL of the medium from each well and add 60 μL of the differentiation medium supplement with 20 μM Y-27632.
96. Incubate the plates in CO_2_ incubator.

#### Day 12: Half differentiation medium changes

**Timing: 0.5 h**

97. Change half of the differentiation medium to achieve a lower concentration of hBMP4 (0.375 nM; final concentration: 13.75 ng/mL).
98. Aspirated 60 μL of the medium from each well and add 60 μL of the differentiation medium supplemented with 20 μM Y-27632.
99. Incubate the plates in CO_2_ incubator.

#### Day 18: Transfer to non-stick petri dishes

**Timing: 1**–**2 h**

100. Transfer the aggregates into a Petri dish.
101. Cut them into 2 to 3 pieces using a V-Lance Knife.
102. Aspirate all the aggregates into 15-mL centrifuges tubes.
103. Remove the supernatant after all the aggregates settle at the bottom of the tubes. Resuspend the aggregates in neural retina medium and transfer them into 9-cm non-stick Petri dishes.

**CRITICAL:** Retinoic acid is more sensitive to light: minimize the light exposure to prevent its isomerization.

**CRITICAL:** Step 103 should be performed in the dark.

#### Long-term culture of neural retinae

**Timing: 0.5 h**

104. Refresh the medium every 5 days and protect the culture from light. Troubleshooting 5

**CRITICAL:** Retinoic acid is more sensitive to light: minimize the light exposure to prevent its isomerization.

**CRITICAL:** Step 143 should be performed in the dark.

## Expected outcomes

**Figure 4:**
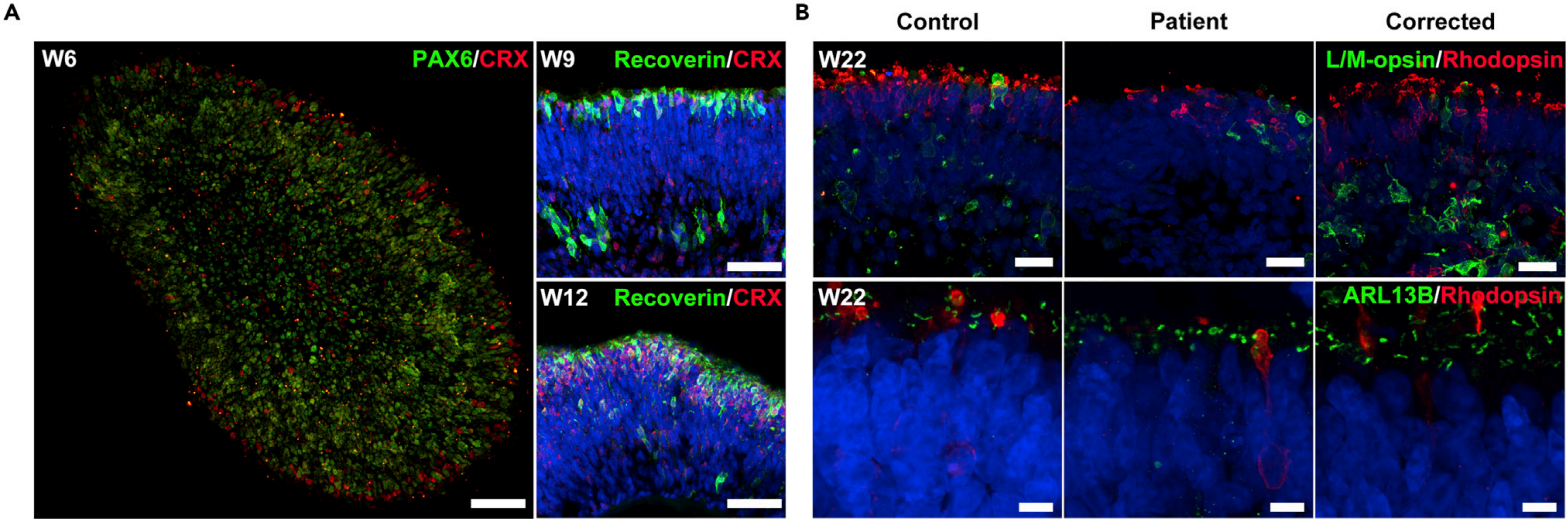
Immunofluorescence of the developing and late organoids Representative confocal images of retinal organoids at different differentiation days. (A) Retinal organoids stained with PAX6 (green), CRX (red), Recoverin (green), and DAPI (blue). Scale bars, 50 μm. (B) Retinal organoids of different groups stained with Rhodopsin (red), L/M-opsin (green), ARL13B (green), and DAPI (blue). Scale bars, 20 μm (top) and 5 μm (bottom). W6, differentiation week 6. W9, differentiation week 9. W12, differentiation week 12. W22, differentiation week 22.

We successfully recapitulated RP predisposed by the *RPGR* mutation, using patient-derived retinae in a dish. The defects in patient hiPSC-derived retinae are consistent with those in their clinical phenotype. Significant defects of photoreceptor and shorted cilium were found in patient retinal organoids. The photoreceptor structure and ciliopathy were rescued by CRISPR-Cas9-mediated correction of *RPGR* mutation (Figure 4). This protocol studied RP *in vitro* utilizing RP patient-derived 3D retinal organoids.

## Limitations

Our protocol includes urinary cell reprogramming, CRISPR-Cas9-mediated genome editing, and retinal organoids differentiation. We not only successfully established hiPSCs from RP patients with the *RPGR* mutation but also achieved mutation correction using the CRISPR-Cas9 technology. The steps of the differentiation of human retinal organoids are based on the previously published protocol (Kuwahara et al., 2015), with slight modifications. Furthermore, retinal organoids derived from patient-specific hiPSCs rebuild the occurrence and development of RP *in vitro*. Thus, they are an ideal model for future disease treatment and drug discovery (Jin et al., 2019).

## Troubleshooting

### Problem 1

Electroporation Efficiency (step 31).

We noted variations among donor urinary cells. The electroporation efficiency can be impacted by the procedures used for urinary cells isolation, maintenance, and the culture environment after electroporation.

### Potential solution

1. Isolate urinary cells as quickly as possible (15–30 min) after collecting the urinary samples.
2. To achieve a high electroporation efficiency, use urinary cells at passages 1–3.
3. The cells grown on feeders after electroporation achieve a higher hiPSC generation efficiency than those grown on Matrigel-based feeder-free conditions (Sugii et al., 2011).

### Problem 2

Loss of pluripotency after hiPSC colony isolation (step 35).

### Potential solution

Isolate only well-separated and hESC-like colonies to ensure a hiPSC state before isolation. Scrape the differentiated cells using pipette tips under the microscope in the biological safety cabinet. Further, isolate the hiPSC colony under the microscope and inoculate it into a new Matrigel-coated dish.

### Problem 3

A Poor hiPSC pluripotency status is observed after thawing (step 70).

### Potential solution

Make sure that the morphology of hiPSCs was good before freezing, as this will influence their pluripotency post-thawing. Moreover, confirm that Y-27632 is added to the medium after thawing and passaging to enhance the viability of the stem cells. Furthermore, passage hiPSCs 2–3 times to adjust the cell status before thawing, and then proceed to the next experiment.

### Problem 4

Differentiation Efficiency (step 85).

The differentiation efficiency varies with different cell lines. This might be due to some genetic or epigenetic factors. Moreover, the experimental procedures and the culture environment can also affect the differentiation efficiency. To improve the differentiation efficiency, we suggest that readers should refer to our recent publication about enhancing the efficiency of the photoreceptor precursor during retinal organoids differentiation (Pan et al., 2020).

### Potential solution

1. Isolate multiple cell lines from the same donor and perform several differentiation times.
2. Fully characterize the hiPSC lines via immunostaining with pluripotency markers, karyotyping, and by analyzing teratoma formation.
3. hiPSCs are sensitive to environmental changes during maintenance: thus, the culture medium should not be changed. Maintain the cell lines for 1–3 passages, if necessary.
4. If death of retinal organoids is observed, reduce it by using the pipette tips.

### Problem 5

Retinal organoids adhere to each other (step 104).

### Potential solution

Gently shake the Petri dish every 2 days during the long-term culture of neural retinae; if necessary, separate the organoids that are stuck together, using a V-Lance Knife under a microscope.

## Resource availability

### Lead contact

Further information and requests for resources and reagents should be directed to and will be fulfilled by the lead contact, Zi-Bing Jin (jinzb502@ccmu.edu.cn).

### Materials availability

The mutant and corrected hiPSC lines generated in this protocol will be made available on request, but we may require a payment and/or a completed Materials Transfer Agreement if there is potential for commercial application.

### Data and code availability

This study did not generate or analyze any datasets or code.

## References

[bib1] Deng W.L., Gao M.L., Lei X.L., Lv J.N., Zhao H., He K.W., Xia X.X., Li L.Y., Chen Y.C., Li Y.P. (2018). Gene correction reverses ciliopathy and photoreceptor loss in iPSC-derived retinal organoids from retinitis pigmentosa patients. Stem Cell Reports.

[bib2] Gao M.L., Lei X.L., Han F., He K.W., Jin S.Q., Zhang Y.Y., Jin Z.B. (2020). Patient-specific retinal organoids recapitulate disease features of late-onset retinitis pigmentosa. Front. Cell Dev. Biol..

[bib3] Jin Z.B., Gao M.L., Deng W.L., Wu K.C., Sugita S., Mandai M., Takahashi M. (2019). Stemming retinal regeneration with pluripotent stem cells. Prog. Retin. Eye Res..

[bib4] Kuwahara A., Ozone C., Nakano T., Saito K., Eiraku M., Sasai Y. (2015). Generation of a ciliary margin-like stem cell niche from self-organizing human retinal tissue. Nat. Commun..

[bib5] Liu H., Zhang Y., Zhang Y.Y., Li Y.P., Hua Z.Q., Zhang C.J., Wu K.C., Yu F., Zhang Y., Su J. (2020). Human embryonic stem cell-derived organoid retinoblastoma reveals a cancerous origin. Proc. Natl. Acad. Sci. U S A.

[bib6] Pan D., Xia X.X., Zhou H., Jin S.Q., Lu Y.Y., Liu H., Gao M.L., Jin Z.B. (2020). COCO enhances the efficiency of photoreceptor precursor differentiation in early human embryonic stem cell-derived retinal organoids. Stem Cell Res. Ther..

[bib7] Sugii S., Kida Y., Berggren W.T., Evans R.M. (2011). Feeder-dependent and feeder-independent iPS cell derivation from human and mouse adipose stem cells. Nat. Protoc..

[bib8] Watanabe K., Ueno M., Kamiya D., Nishiyama A., Matsumura M., Wataya T., Takahashi J.B., Nishikawa S., Nishikawa S.I., Muguruma K. (2007). A ROCK inhibitor permits survival of dissociated human embryonic stem cells. Nat. Biotechnol..

[bib9] Zhou T., Benda C., Dunzinger S., Huang Y., Ho J.C., Yang J., Wang Y., Zhang Y., Zhuang Q., Li Y. (2012). Generation of human induced pluripotent stem cells from urine samples. Nat. Protoc..

